# Dr. Haxby's Reply to the Editors

**Published:** 1815-06

**Authors:** John Haxby

**Affiliations:** Pontefract


### *For the Medical and Physical Journal.*

### Dr. Haxby's *Reply to the Editors.*

THE favourable manner in which my communication has been received, demands my grateful acknowledgments and ready compliance with any request you may think right to propose.

I have no doubt that the dyspnœa was, till within a fortnight of my patient's death, exclusively occasioned by the continued pressure of the aneurismal sac on the trachea; the difficulty of breathing being afterwards increased by the infarction of the lungs, consequent on inflammation. There appeared no impediment to the function of the œsophagus, either in deglutition or vomiting.

I regret that it did not occur to me to make the experiment of pressing the air back from the vesicles into its natural cells, though I think it more than probable that the communication was, in many places, pervious, from the unequal prominence of the vesicles, and, if I recollect right, from a sensible elevation of them when pressure was accidentally made on the lungs; besides, I can in no other way account for the particular sound which was heard during the act of laborious expiration, but by supposing that the compressed air, not finding a ready exit from the cells through the trachea, was forced back into the vesicular caverns, exhibiting a species of ventriloquism, and occasioning, by its want of renovation, the extraordinary anxiety at those times complained of.

Why the crasis of the spleen was so entirely broken down, while the structure of the liver, kidnies, and all the other abdominal viscera, was so firm and healthy, I cannot satisfactorily explain. Had it depended on the de-oxigenated state of the blood loosely coagulated, the effect would have been more general. The same reasoning will apply to the partial sphacelation of the lungs, which, as well as the effusion, I have no doubt, was induced by previous inflammation of these organs, indicated by the quickness of the pulse, by increased heat, thirst, &c. which took place only about a fortnight before the death of the patient.

In addition to Mr. Rowe's remarks on a case of supposed confluent varicella, I beg to observe, that I have met with two or three instances of a copious pustular eruption subsequent to vaccination, where the stages were intermediate in duration between varicella and variola, and where the vesicles contained a purulent fluid, and were succeeded by a few distinct *foveæ*. I do not remember that any person was inoculated from the matter of these pustules, as it was concluded they were merely the chicken-pox. Is the observation confirmed, that varicella, following vaccine, is more severe in proportion to the proximity of its succession?

I am happy to find, by one of your preceding Numbers, that Dr. Kinglake's attention has been called to a point of great importance in medical practice, which still divides the opinion of a great portion of the faculty in this part of the country. I allude to the contagious nature of phthisis, or of some varieties of this disorder. I do not profess myself an advocate for its being infectious; though, from a proper degree of caution, I prevent, as much as I am able, unnecessary exposure of the patient's friends to the inhalation of air which has been returned from the lungs in a state of ulceration. I trust Dr. K. will prosecute this interesting enquiry, and invite the assistance of the members of the profession.

*Pontefract;* JOHN HAXBY, M.D.
*April* 21, 1815.

